# Reading assessment in ADHD and dyslexia in Brazilian teenagers

**DOI:** 10.1192/j.eurpsy.2023.1496

**Published:** 2023-07-19

**Authors:** A. G. Seabra, G. R. Brito, L. R. R. Carreiro

**Affiliations:** Postgraduate Program in Developmental Disorders, Mackenzie Presbyterian University, Sao Paulo, Brazil

## Abstract

**Introduction:**

Attention Deficit Hyperactivity Disorder (ADHD) and Dyslexia are among the most frequent developmental disorders in school-aged students, and both often cause an impact on scholar reading performance. Therefore, it is fundamental to trace the differential profile in reading performance in such diagnoses. Competent reading occurs through the interaction of several cognitive processes, such as decoding, fluency, and oral and reading comprehension, that should be assessed in an evaluation.

**Objectives:**

The study aimed to characterize the performance of students with ADHD and dyslexia.

**Methods:**

We assessed 25 adolescents, aged between 11 and 14 years old, from 6th to 9th year of middle school of public and private schools in Brazil, divided into two groups: the group with ADHD (16 students) and the group with dyslexia (9 students). The diagnoses were established by a multidisciplinary center and there were no comorbidities for any case. The instruments used were: Comprehension Test of Words and Pseudowords II (TCLPP II) to assess decoding (indicate if the word is correct or incorrect); Reading Fluency Test (TFL) to assess fluency in single words and in text reading; Cloze Reading Comprehension Test (TCCL) to measure reading comprehension; and the WISC vocabulary subtest to assess auditory comprehension.

**Results:**

Non-parametric analyzes revealed statistically significant differences in measures of textual comprehension, especially in the tasks that involved decoding and fluency processes, evidencing superior performance of the group with ADHD in these tests. Participants with dyslexia had a significantly higher performance in relation to the number of word omissions, that is, they had lower omission errors. There was no significant difference between groups in auditory comprehension.

**Conclusions:**

A differential profile was found in reading performance, consistent with the cognitive deficits classically pointed out in the literature for each diagnosis: phonological deficits in dyslexia, with problems in decoding and fluency; and attentional deficits in ADHD, with omission errors. In the comprehension measures, dyslexic group had significant lower performance than ADHD in the Cloze Reading Comprehension Test, but there was no difference in the Vocabulary subtest-WISC. An explanatory hypothesis is that, to understand the text, it is necessary to recognize the words previously, whereas, in the WISC, it is not necessary to read, since the questions are oral. These results corroborate the hypothesis that deficits in reading comprehension in dyslexia are more related to difficulties in word recognition and fluency skills than in general listening comprehension.

Financial support: CAPES Proex [grant 0426/2021, no. 23038.006837/2021-73]; CNPq [grant 310845/2021-1]

**Disclosure of Interest:**

None Declared

